# Supplementary material for: Evidence for widespread alterations in cortical microstructure after 32 h of sleep deprivation
Source: Transl Psychiatry. 2022 Apr 14;12:161. doi: 10.1038/s41398-022-01909-x (PMC9010475; doi:10.1038/s41398-022-01909-x)

Supplementary Material

Evidence for widespread alterations in cortical microstructure after 32 hours of sleep deprivation

Authors:

Irene Voldsbekk[a,b,c,*], Atle Bjørnerud [a,c,d], Inge Groote[c], Nathalia Zak [b,f], Daniel Roelfs [b,g], Ivan I. Maximov [a,b,h], Oliver Geier [f], Paulina Due-Tønnessen[i], Erlend Bøen[j], Yvonne S. Kuiper[f], Lise-Linn Løkken[f], Marie Strømstad[a], Taran Y. Blakstvedt[a], Bjørn Bjorvatn[k,l], Ulrik F. Malt[g], Lars T. Westlye[a,b,m], Torbjørn Elvsåshagen [b,g,n], Håkon Grydeland[a,o,*]

[a] Department of Psychology, University of Oslo, Oslo, Norway.

[b] Norwegian Centre for Mental Disorders Research (NORMENT), Oslo University Hospital, Oslo, Norway.

[c] Computational Radiology and Artificial Intelligence (CRAI), Division of Radiology and Nuclear Medicine, Oslo University Hospital, Oslo, Norway.

[d] Department of Physics, University of Oslo, Oslo, Norway.

[e] Department of Radiology, Vestfold Hospital Trust, Tønsberg, Norway.

[f] Department of Diagnostic Physics, Division of Radiology and Nuclear Medicine, Oslo University Hospital, Oslo, Norway.

[g] Institute of Clinical Medicine, University of Oslo, Oslo, Norway.

[h] Department of Health and Functioning, Western Norway University of Applied Sciences, Bergen, Norway.

[i] Division of Radiology and Nuclear Medicine, Oslo University Hospital, Oslo, Norway.

[j] Psychosomatic and CL Psychiatry, Division of Mental Health and Addiction, Oslo University Hospital, Oslo, Norway.

[k] Department of Global Public Health and Primary Care, University of Bergen, Bergen, Norway.

[l] Norwegian Competence Center for Sleep Disorders, Haukeland University Hospital, Bergen, Norway.

[m] KG Jebsen Centre for Neurodevelopmental Disorders, University of Oslo, Oslo, Norway.

[n] Department of Neurology, Oslo University Hospital, Oslo, Norway.

[o] Center for Lifespan Changes in Brain and Cognition, Department of Psychology, University of Oslo, Norway.

1 Change in Euler number and hydration in each group

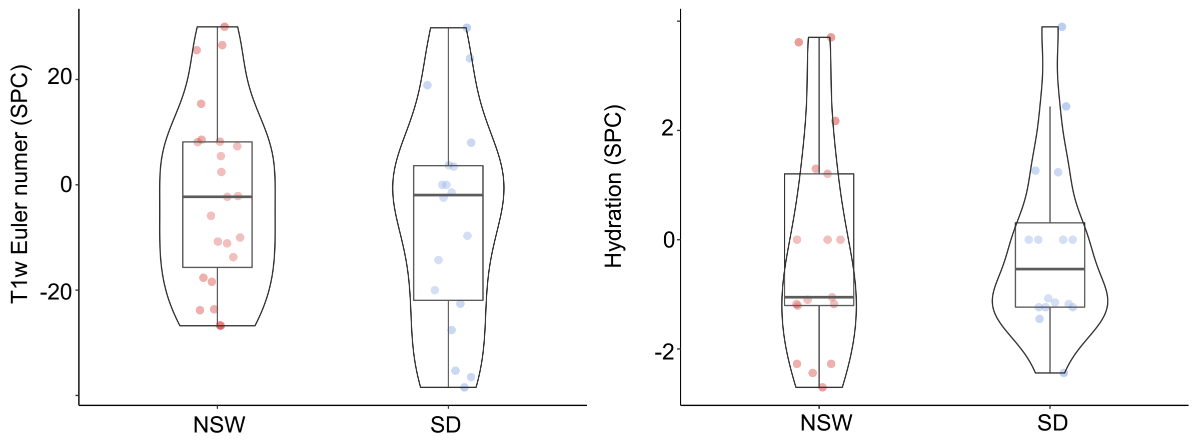

2 Main analysis rerun excluding one participant in the sleep deprivation group

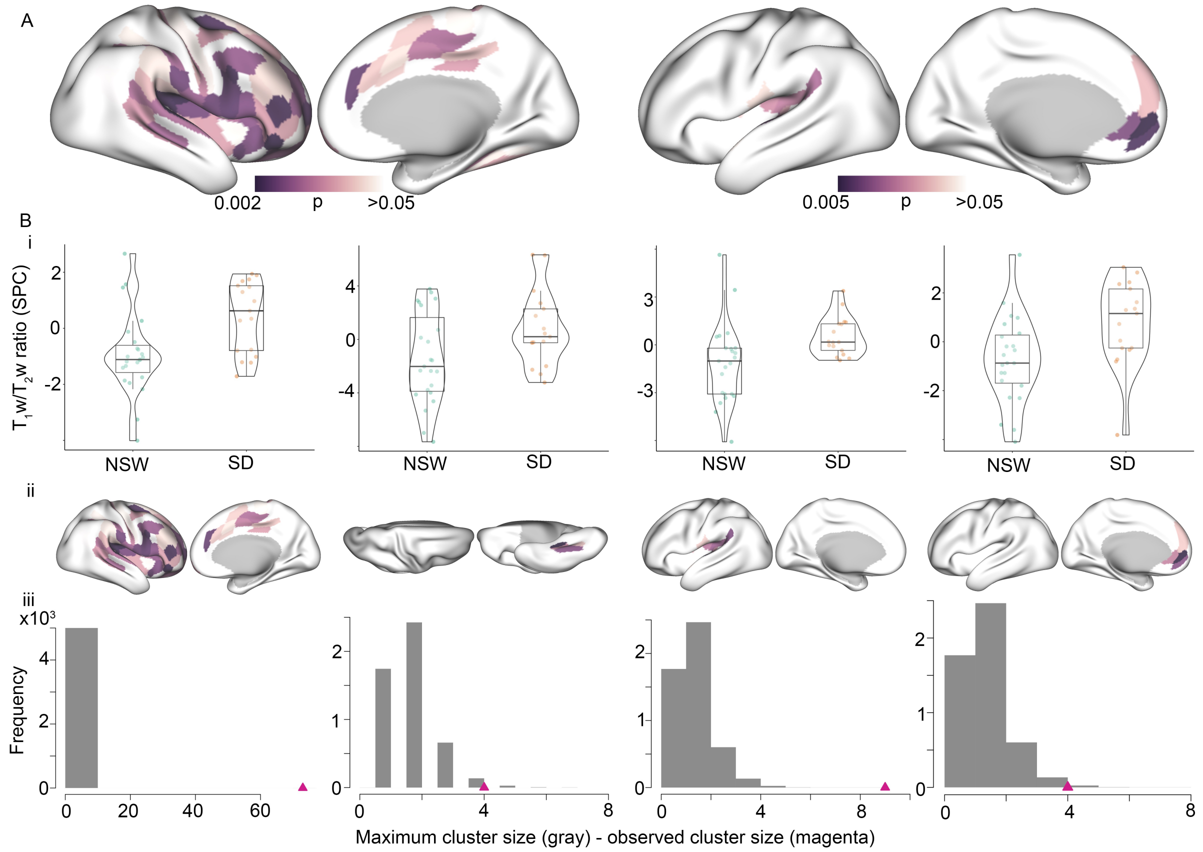

3 Difference in sleepiness and attention across the 32 hours of the study

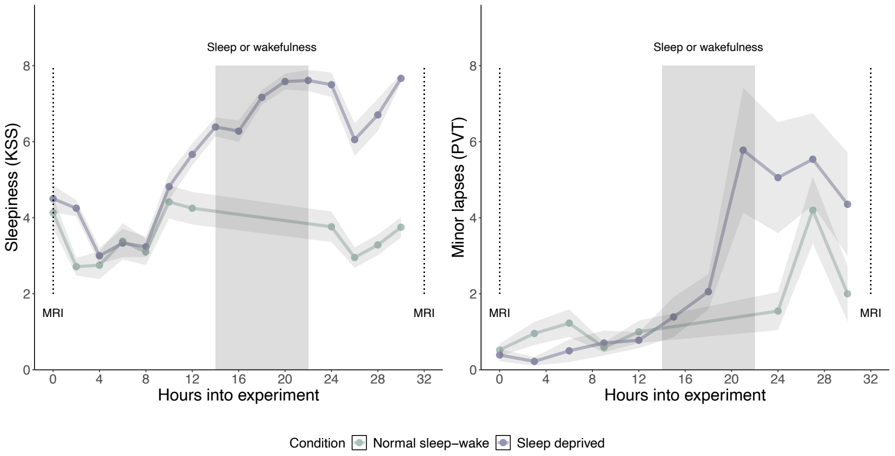

4 Distribution of change in sleepiness and attention from TP1 to TP2

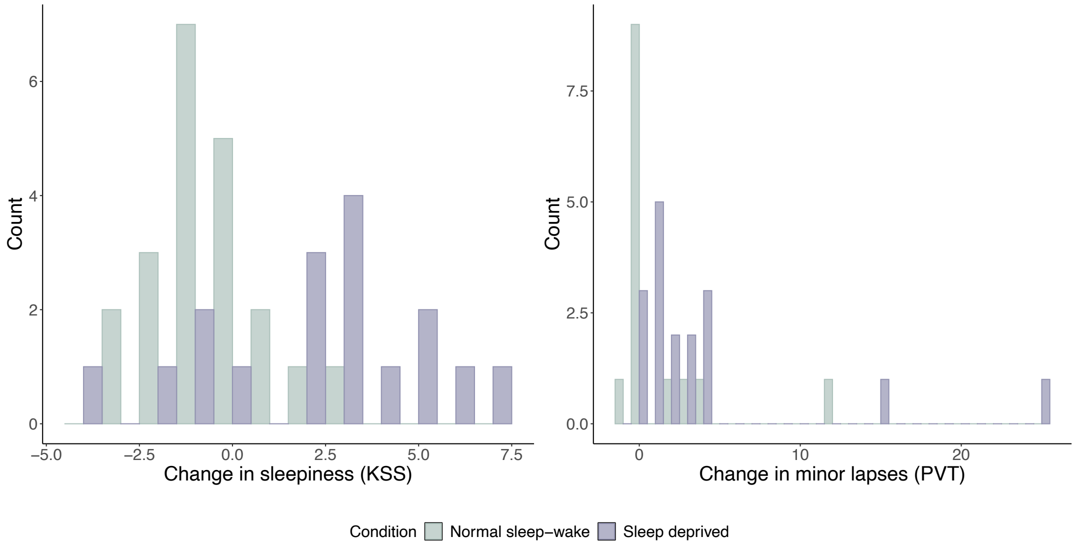

5 Alternative measures of attention from the PVT: group differences

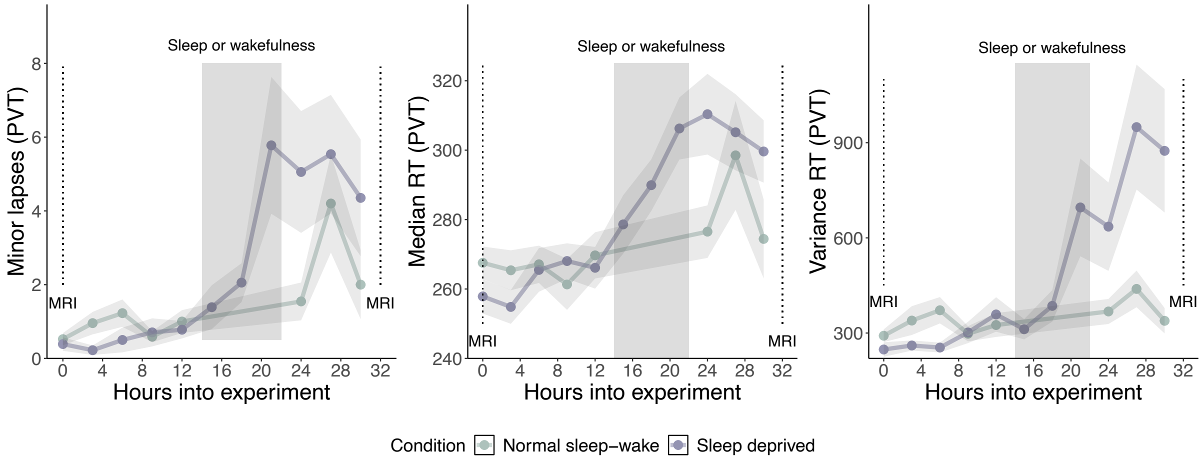

6 Uncorrected correlations between changes in T_1w/T_2w ratio and sleepiness

RH #1

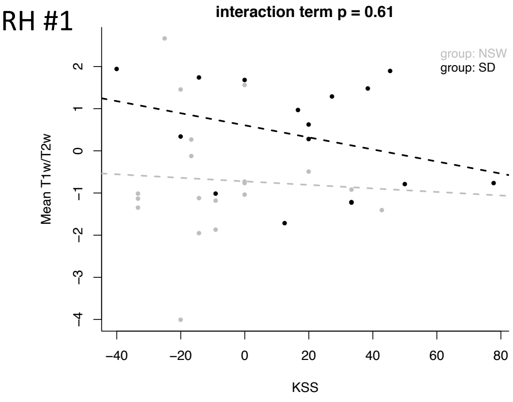

RH #2

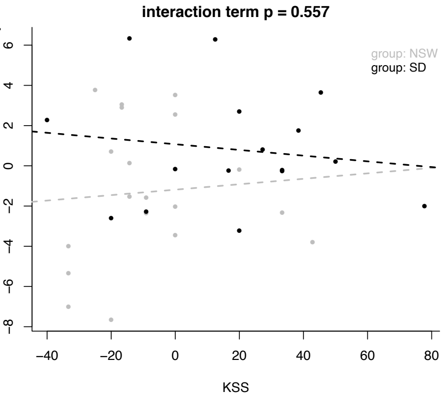

LH #1

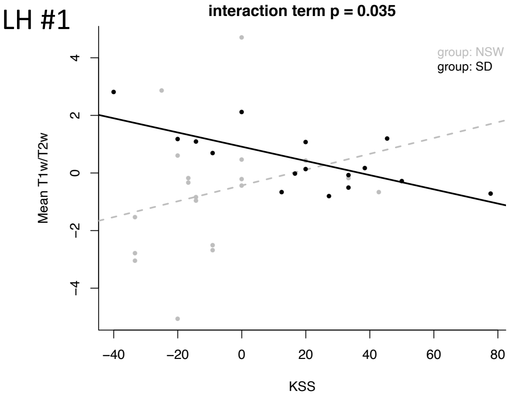

LH #2

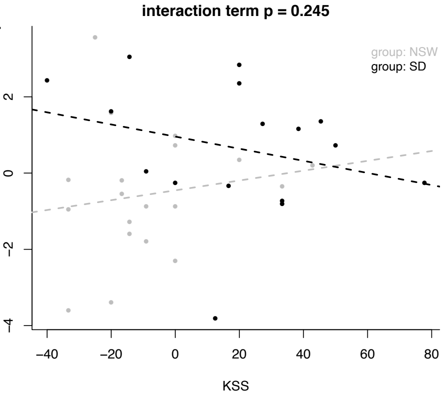

7 Uncorrected correlations between changes in T_1w/T_2w ratio and lapses in attention

RH #1

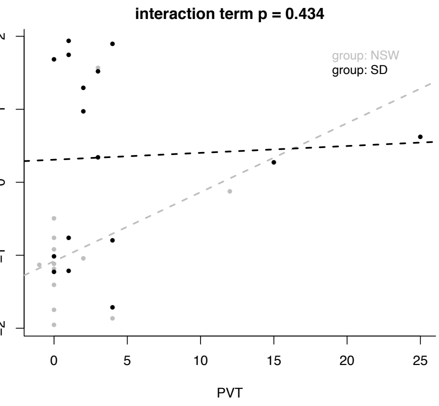

RH #2

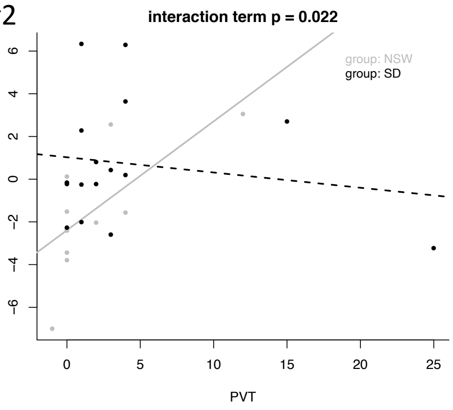

LH #1

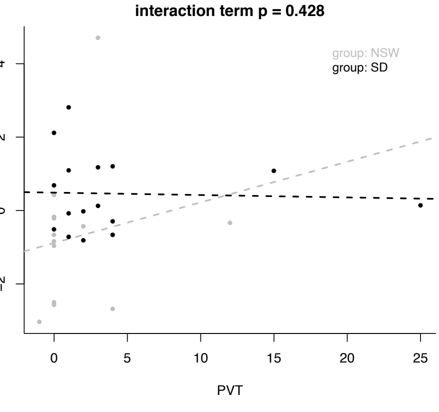

LH #2

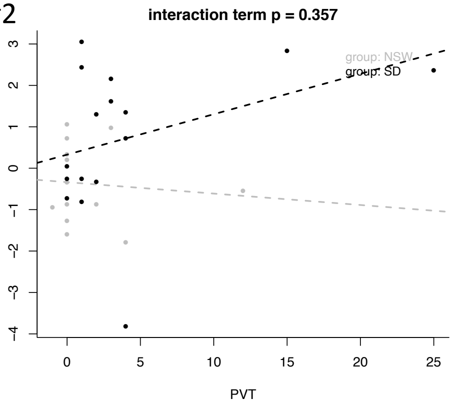

Supplement: Supplementary file 1 — Supplementary Material [file 41398_2022_1909_MOESM1_ESM.pdf]
